# Distinct diagnostic trajectories in NBAS‐associated acute liver failure highlights the need for timely functional studies

**DOI:** 10.1002/jmd2.12280

**Published:** 2022-03-15

**Authors:** Lauren S. Akesson, Rocio Rius, Natasha J. Brown, Jeremy Rosenbaum, Sarah Donoghue, Michael Stormon, Charmaine Chai, Esmeralda Bordador, Yiran Guo, Hakon Hakonarson, Alison G. Compton, David R. Thorburn, Sumudu Amarasekera, Justine Marum, Alisha Monaco, Crystle Lee, Belinda Chong, Sebastian Lunke, Zornitza Stark, John Christodoulou

**Affiliations:** ^1^ Victorian Clinical Genetics Services, Murdoch Children's Research Institute Royal Children's Hospital Melbourne Victoria Australia; ^2^ Department of Paediatrics University of Melbourne Melbourne Victoria Australia; ^3^ SA Pathology SA Health Adelaide SA Australia; ^4^ School of Biomedicine, Faculty of Medicine, Dentistry and Health Sciences University of Adelaide Adelaide Australia Australia; ^5^ Brain and Mitochondrial Research Group Murdoch Children's Research Institute, Royal Children's Hospital Melbourne Victoria Australia; ^6^ Department of Gastroenterology Royal Children's Hospital Melbourne Victoria Australia; ^7^ Department of Metabolic Medicine Royal Children's Hospital Melbourne Victoria Australia; ^8^ Department of Gastroenterology Children's Hospital Westmead Sydney New South Wales Australia; ^9^ Discipline of Child & Adolescent Health, Sydney Medical School University of Sydney Sydney New South Wales Australia; ^10^ Center for Applied Genomics Children's Hospital of Philadelphia Philadelphia Pennsylvania USA; ^11^ Center for Data‐Driven Discovery in Biomedicine Children's Hospital of Philadelphia Philadelphia Pennsylvania USA; ^12^ Department of Pediatrics, Perelman School of Medicine University of Pennsylvania Philadelphia Pennsylvania USA; ^13^ Department of Pathology University of Melbourne Melbourne Victoria Australia

**Keywords:** functional genomics, genome sequencing, pediatrics, rapid genomic sequencing, recurrent acute liver failure, variant classification

## Abstract

Variants of uncertain significance (VUS) are commonly found following genomic sequencing, particularly in ethnically diverse populations that are underrepresented in large population databases. Functional characterization of VUS may assist in variant reclassification, however these studies are not readily available and often rely on research funding and good will. We present four individuals from three families at different stages of their diagnostic trajectory with recurrent acute liver failure (RALF) and biallelic *NBAS* variants, confirmed by either trio analysis or cDNA studies. Functional characterization was undertaken, measuring NBAS and p31 levels by Western blotting, demonstrating reduced NBAS levels in two of three families, and reduced p31 levels in all three families. These results provided functional characterization of the molecular impact of a missense VUS, allowing reclassification of the variant and molecular confirmation of *NBAS*‐associated RALF. Importantly, p31 was decreased in all individuals, including an individual with two missense variants where NBAS protein levels were preserved. These results highlight the importance of access to timely functional studies after identification of putative variants, and the importance of considering a range of assays to validate variants whose pathogenicity is uncertain. We suggest that funding models for genomic sequencing should consider incorporating capabilities for adjunct RNA, protein, biochemical, and other specialized tests to increase the diagnostic yield which will lead to improved medical care, increased equity, and access to molecular diagnoses for all patients.

**SYNOPSIS**

Access to rapid genomic and adjunct functional studies will improve clinical care of patients with NBAS‐associated recurrent acute liver failure and other monogenic conditions.

## 1 INTRODUCTION

Rapid genomic sequencing is revolutionizing management of seriously ill infants and children, with the potential to confirm a molecular diagnosis within a few days with a high degree of clinician‐reported clinical utility.^1^, ^2^ However, many patients receive uncertain results due to the lack of evidence supporting pathogenicity of rare or novel variants. Reclassification of variants of uncertain significance (VUS) in genes that are phenotypically concordant with the clinical presentation remains challenging in all clinical settings, but particularly when a rapid diagnosis is needed to guide clinical management. Functional characterization of VUS may be required to determine pathogenicity and allow a definitive molecular diagnosis, however these studies are often not readily available, are time consuming, and rely on research funding and good will. Improved access to supplementary adjunct studies allows reclassification of splicing VUS in a clinically meaningful timeframe,^3^ a model that may be applied to functional genomics studies.

Biallelic *NBAS* variants were first associated with short stature, optic nerve atrophy, and Pelger‐Huet anomaly (SOPH, MIM 614800) in 2010,^4^ followed by reports of an association between biallelic *NBAS* variants and recurrent acute liver failure (RALF) or Infantile liver failure syndrome 2 in 2015 (ILFS2, MIM 616483).^5^ Since that time, over 100 individuals with biallelic *NBAS* variants and RALF have been reported, with a mutational spectrum including loss of function, missense, and deep intronic variants.^6^ The neuroblastoma amplified sequence (NBAS) protein is a component of the endoplasmic reticulum tethering complex involved in retrograde Golgi to endoplasmic reticulum transport, interacting with a number of intracellular proteins including p31.^5^ Disease‐causing pathogenic *NBAS* variants may result in decreased NBAS protein levels, however some variants, particularly biallelic missense variants, may be associated with normal NBAS protein levels despite altered NBAS function.^7^ Previous studies have demonstrated that disease‐causing *NBAS* variants result in decreased p31 protein levels.^5^, ^7^

We present clinical, genomic and functional data from three families with biallelic variants in *NBAS*. Our functional data assisted reclassification of one VUS to likely pathogenic, and two likely pathogenic variants to pathogenic, thus confidently securing molecular diagnoses in these families.

## 2 METHODS

### 2.1 Case descriptions

#### 2.1.1 Individual A

An 18‐month‐old female presented with RALF. At 8 months, she was admitted to hospital with vomiting and fever and was found to have alanine aminotransferase (ALT) activity of 739 IU/L (normal range 4–45), which resolved with supportive treatment. Coagulation studies were not performed. At age 11 months, she developed symptoms suspicious for sepsis and was found to have hypoglycemia, hypernatremia, lactic acidosis, coagulopathy, and liver derangement (ALT 9683 IU/L, AST 14760 IU/L, international normalized ratio (INR) 9.9 (normal range 0.8–1.2)). She was treated with intravenous fluids, antibiotics, antivirals, fresh frozen plasma, vitamin K and corticosteroids, and her symptoms resolved over a few days. Adenovirus was isolated from a stool sample. At 18 months, she developed symptoms of a viral respiratory infection and was found to have an ALT of 11 524 IU/L and lactate dehydrogenase (LDH) of 20 823 U/L (normal range 180–300) accompanied by a coagulopathy with an INR of 9.1 and unexplained hypoglycemia. There was no history of an ischemic insult or exposure to toxins. A liver biopsy was performed for diagnostic purposes, as investigations for metabolic and infective causes of liver failure were non diagnostic. Results were nonspecific with severe diffuse acute pauci‐inflammatory hepatocellular injury, with zonal hepatocyte necrosis. Extensive metabolic investigations were nondiagnostic. There was no history of an ischemic insult or exposure to toxins.

She has subsequently experienced three further episodes requiring hospital admission (at 2 years 6 months, 2 years 11 months, and 3 years 2 months), all in the context of viral precipitants. The peak INR at these presentations was 2.0, 1.0, and 2.7, respectively. All episodes resolved with supportive therapy. Apart from these six episodes, she remains otherwise well with normal intervening blood test results. She has normal growth and neurodevelopment with no craniofacial dysmorphism. There is no family history of liver disease.

#### 2.1.2 Individuals B1 and B2

Individual B1 had an antenatal history of intrauterine growth restriction from 32 weeks of gestation. Physical examination showed rhizomelia and large fontanelles. Although reported to be irritable, his neurodevelopment was normal prior to the onset of viral illness with elevated liver enzymes at age 7 months. Hepatitis A and B serology and blood lactate and pyruvate at this time were normal. Subsequently, he had recurrent episodes characterized by initial signs of a viral illness and then deterioration with prostration, drowsiness, and markedly elevated liver enzymes. However, coagulopathy was not documented. Fasting studies showed a blood glucose level of 2.5 mmol/L with B‐hydroxybutyrate increased markedly, reaching 9.1 mmol/L. Succinyl‐CoA transferase and acetoacetyl‐CoA thiolase activities in cultured skin fibroblasts were normal. The episodes would resolve in a few days with liver enzyme levels returning to baseline. He had several admissions to hospital until he died at age of 14 months. Respiratory chain enzymology in skin fibroblasts and skeletal muscle were normal, while liver homogenate results were deficient for complex II in both a biopsy and perimortem sample (17% and 19% of control mean relative to protein and 23% and 16% of control mean when expressed relative to citrate synthase, respectively). His female sibling, individual B2, had a similar presentation with episodic severe RALF with intercurrent viral infections. Unlike her brother, she had no documented hypoglycemia, ketosis or metabolic acidosis. The episodes would resolve in a few days with the liver function enzymes returning to baseline. Fibroblast fatty acid oxidation screen, and specific assays for Long‐chain L‐3‐hydroxyacyl‐CoA dehydrogenase deficiency and short chain l‐3‐hydroxyacyl‐CoA dehydrogenase were normal. She died aged 2 years 9 months during an acute episode. Respiratory chain enzymology in liver was borderline low for complex III and normal for all other enzymes.

#### 2.1.3 Individual C

Individual C has been previously described.^8^ Briefly, a 13‐month old female presented with RALF, usually prompted by febrile infections. They were characterized by transaminases being in the tens of thousands, severe coagulopathy with INR up to 9.1 (RR 1.0–1.2), and lactic acidosis with or without hypoketotic hypoglycemia. Urgent liver transplantation was considered. However, with supportive dextrose parenteral infusions, the episodes resolved within 2 weeks and the frequency of RALF reduced with age, with her last episode being at 6 years. Between episodes, laboratory, and imaging findings were normal. Respiratory chain enzymology in liver was normal, while muscle results demonstrated a possible complex II + III deficiency (22% of normal relative to protein, 14% relative to citrate synthase, and 15% relative to complex II). She was treated with coenzyme Q and L‐carnitine. Despite these episodes, her growth and neurodevelopment were normal.

### 2.2 Genomic sequencing

Genomic sequencing was performed by clinically accredited laboratories at the Victorian Clinical Genetics Services (Melbourne, Australia) (individual A), The Center for Applied Genomics (The Children's Hospital of Philadelphia, USA) (individuals B1 and B2), and Kinghorn Centre for Clinical Genomics (Garvan Institute, Sydney, Australia) (individual C), using genome sequencing (GS) or exome sequencing (ES) techniques. For individual A, GS was performed using massively parallel sequencing (MPS) (Nextera DNA Flex Library Prep kit (Illumina Sequencers, San Diego, CA, USA) with a mean target coverage of 30×, and a minimum of 90% of bases sequenced to at least 10×. Data were processed, including read alignment to the reference genome (GRCh38) and variant calling, using Cpipe^9^ or a functionally equivalent analysis with the Illumina Dragen System (Illumina). Variant analysis and interpretation within the selected target region (RefSeq genes ± 1 kb) was performed using Alissa Interpret (Agilent). Variants were annotated against all RefSeq gene transcripts and reported in accordance with HGVS nomenclature.^10^ Copy number variants (CNV) were screened for using an internal CNV detection tool.^11^ All reported CNVs were orthogonally validated unless otherwise stated. Individuals B1 and B2 had ES performed as previously described.^12^ In brief, libraries were constructed and sequenced on an Illumina HiSeq 2000 Sequencing System (Illumina, San Diego, CA, USA). Reads were aligned to reference genome (UCSC hg19), using Burrows–Wheeler alignment (BWA).^13^ Genomic variants were then called, including single nucleotide variants (SNVs) and small insertions/deletions (indels), by an integrated in‐house pipeline, using Genome Analysis Tool Kit (GATK, v1.4).^14^ In individual C, trio GS was also performed as previously described,^8^, ^15^ and samples were sequenced on an Illumina HiSeq × Ten sequencer (Illumina, Software, v3.0.29.0). Reads were aligned to the b37d5 reference genome using BWA,^13^ sorted using Novosort v.1.03.01 (Novocraft Technologies), then realigned and recalibrated using GATK v.3.3. Variants were identified using GATK HaplotypeCaller v.3.3.^16^ Databases were imported into SEAVE, which was used to perform variant filtration and prioritization.^17^

Curation of variants was phenotype‐driven with pre‐curated or custom gene lists used for variant prioritization. Variant classification was based on modified ACMG/AMP guidelines.^18^

### 2.3 Cell culture and Western blotting

All fibroblast cell lines were tested for *Mycoplasma*. Skin fibroblasts were cultured at 37°C in 5% CO_2_ in Dulbecco's Modified Eagle Medium (DMEM) 3.7 g/L NaHCO_3_ (HyClone) supplemented with 10% Fetal Bovine Serum (FBS) and 1% penicillin/streptomycin. Protein was extracted from cultured fibroblasts by resuspending in radioimmunoprecipitation assay (RIPA) buffer with protease inhibitor cocktail (Roche), then sonicated and incubated on ice for 30 min before centrifugation at 18000*g* for 20 min at 4°C. Total protein concentration was determined with the Pierce Bicinchoninic acid (BCA) kit (ThermoFisher) following the manufacturer's protocol. Protein lysates containing 20 μg of protein samples were analyzed by SDS‐PAGE Western blot (BioRad system) using primary antibodies against NBAS (1:500, ThermoFisher, PA5‐103963), USE1 (p31) (1:250 Sigma Aldrich, HPA026851) and GAPDH (1:10 000; Sigma Aldrich, G9545), and detected by anti‐rabbit IgG secondary‐horseradish peroxidase (HRP) conjugated antibody (1:5000, Cell Signaling Technology, #7074S) using Enhanced chemiluminescence reagents (ECL) (GE Healthcare). Protein band intensities were quantified using ImageJ software and normalized to GAPDH.

### 2.4 Cycloheximide treatment and cDNA studies

To inhibit nonsense‐mediated decay, Case B1 and control fibroblasts at ~70% confluency were treated with 100 ng/μL cycloheximide (Sigma‐Aldrich, #C6255) for 24 h before harvesting.^19^ RNA was extracted using the miRNeasy Mini kit (Qiagen) and cDNA was synthesized using the Superscript III kit (Thermo Fisher Scientific) following the manufacturer's protocol. cDNA was amplified by polymerase chain reaction (PCR) using primers designed to sequence *NBAS* exons 12–15 (5′ACTGAGCATCTGGGCGATTC3′, 5′AGTAATGGTTCGTGGGCGTT3′) and 25–30 (5′GCCTACCAGTGGATGGTTCC3′, 5′CTGGCAGCCAAAACCAAGTC 3′).

## 3 RESULTS

### 3.1 Genomic sequencing

#### 3.1.1 Individual A

Ultra‐rapid trio GS, including analysis of the mitochondrial genome, was performed during an acute episode of RALF, identifying compound heterozygous missense variants in *NBAS* with results available in 71 h. The paternally inherited variant, NM_015909.3(NBAS):c.2951T>G; p.(Ile984Ser), was classified as likely pathogenic according to modified ACMG/AMP criteria.^18^ The variant was present in a large population database at a frequency of <0.01% (3 heterozygotes, 0 homozygotes) (gnomAD v2.1.1,^20^ https://gnomad.broadinstitute.org/ [accessed 15/11/2019]). It has been previously reported in individuals with RALF.^5^, ^6^ Computational evidence for pathogenicity is conflicting with uninformative conservation (100 vertebrates, UCSC,^21^ https://genome.ucsc.edu/ [accessed 15/11/2019]). There is a large physicochemical difference between isoleucine and serine (Grantham Distance of 142).^22^ The variant is located in a well‐established functional domain (Sec39). The maternally inherited variant, NM_015909.3(NBAS):c.406A>G; p.(Arg136Gly), was classified as a VUS according to modified ACMG/AMP criteria.^18^ The variant was present in a large population database at a frequency of <0.01% (1 heterozygote, 0 homozygotes) (gnomAD v2.1.1,^20^ https://gnomad.broadinstitute.org/ [accessed 15/11/2019]). It has not been previously reported in association with disease. Computational tools predict a deleterious effect of the variant on protein function, and the variant is highly conserved (100 vertebrates, UCSC,^21^ https://genome.ucsc.edu/ [accessed 15/11/2019]). There is a large physicochemical difference between arginine and glycine (Grantham Distance of 125).^22^ The variant is located in the β‐propeller domain.^23^ The phenotype of individual A was considered to be a strong and specific match for the gene of interest (*NBAS*).

#### 3.1.2 Individuals B1 and B2

**FIGURE 1:**
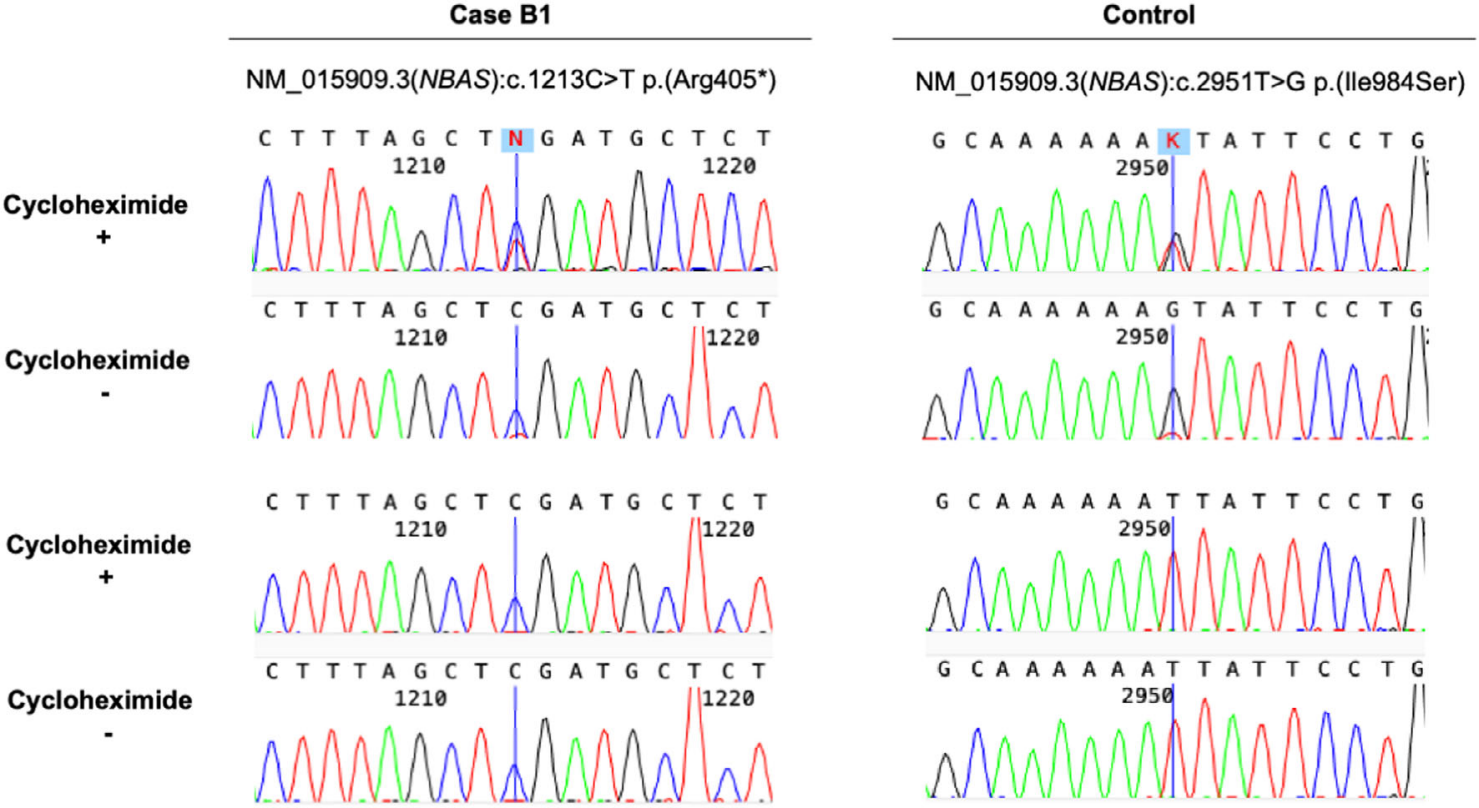
cDNA studies in Individual B1. The NM_015909.3(*NBAS*):c.2951T>G, p.(Ile984Ser) variant appeared heterozygous when amplifying the cDNA generated from cells with cycloheximide treatment and as homozygous when amplifying the cDNA generated from cells grown without cycloheximide, suggesting that the allele with the p.(Arg405*) is *in trans* and largely degraded by nonsense‐mediated decay (NMD)

Exome sequencing was undertaken decades after death from stored DNA of individual B1, identifying two heterozygous missense variants in *NBAS*: the NM_015909.3(*NBAS*):c.2951T>G; p.(Ile984Ser) likely pathogenic variant described in individual A, and a novel variant, NM_015909.3(*NBAS*):c.1213C>T; p.(Arg405*), classified as pathogenic according to modified ACMG/AMP criteria. The p.(Arg405*) variant was absent from a large population database (gnomAD v2.1.1^20^ [accessed 07/10/2020]). It has not been previously reported in association with clinical disease, however many upstream and downstream variants also resulting in a premature termination codon have been reported.^6^ cDNA studies confirmed the transcript to be subject to degradation by nonsense mediated decay (NMD) (Figure 1). The phenotype of individual B1 was considered to be a strong and specific match for the gene of interest. Sanger sequencing of his affected sibling B2 identified both variants. Parental DNA was not available for segregation, however cDNA studies in B1 confirmed compound heterozygosity of the variants (Figure 1).

#### 3.1.3 Individual C

ES, GS and cDNA studies were performed, identifying biallelic variants in *NBAS* as previously described.^8^ These variants were previously described in a case report of individual C.^8^ Briefly, the maternally inherited variant NM_015909.4(*NBAS*):c.2617C>T p.(Arg873Trp) was classified as likely pathogenic according to modified ACMG/AMP criteria. The variant was present in a large population database at a frequency of <0.01% (2 heterozygotes, 0 homozygotes) (gnomAD v2.1.1,^20^ [accessed 12/10/2020]). Computational evidence for pathogenicity is consistently predicted to be damaging and the variant is highly conserved. There is a large physicochemical difference between arginine and tryptophan (Grantham Distance of 101).^22^ The variant is located in a well‐established functional domain (Sec39). The paternally inherited variant, NM_015909.4(*NBAS*):c.2423+404G>C, is a deep intronic variant and was classified as likely pathogenic. The variant was present in a large population database at a frequency of <0.01% (2 heterozygotes, 0 homozygotes) (gnomAD v2.1.1,^20^ [accessed 12/10/2020]). cDNA studies confirmed a splicing defect leading to the inclusion of a pseudo‐exon and premature termination codon leading to nonsense‐mediated RNA decay (NMD).^8^ Many upstream and downstream variants also resulting in NMD have been reported.^6^ The phenotype of individual C was considered to be a strong and specific match for the gene of interest.

### 3.2 Patient fibroblasts display decreased levels of p31

**FIGURE 2:**
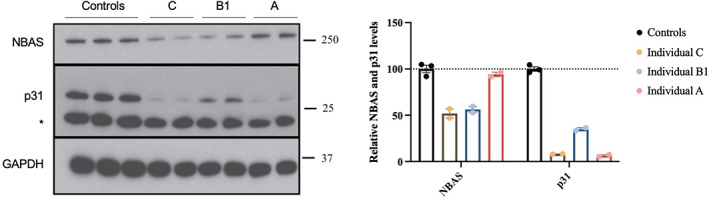
NBAS and p31 protein expression in fibroblasts. Representative Western blot and densitometry analysis suggest NBAS protein levels of 40%–50% of control mean in individual C and B1, and p31 levels of 5% in individual A, 35% in individual B1 and 10% in individual C relative to control mean, GAPDH was used as a protein loading control

Western blotting of fibroblast cell lysates showed decreased NBAS protein expression in individual B1 and individual C compared to control cells (50% and 60% relative reduction, respectively). However, the NBAS protein levels in individual A who carried biallelic missense variants, were similar to controls. There was an overall reduction in p31 levels in all cases compared to controls (Figure 2).

### 3.3 Variant reclassification

The missense VUS (p.(Arg136Gly)) identified in individual A was reviewed by the diagnostic genomic laboratory following completion of the adjunct functional studies, and reclassified as Likely Pathogenic. The missense variant identified in individual A and B, previously classified as Likely Pathogenic (p.(Ile984Ser)), was upgraded to Pathogenic, based on the functional data. Likewise, the p.(Arg873Trp) and NM_015909.4(*NBAS*):c.2423+404G>C variants identified in individual C could be reclassified to Pathogenic.

## 4 DISCUSSION

This study describes three families who received a molecular diagnosis of *NBAS*‐associated RALF, including one individual where adjunct studies were used to reclassify a VUS, highlighting the importance of access to timely functional studies after identification of putative variants, and the importance of selecting appropriate orthogonal analyses to reclassify VUS.

This study raises several important aspects. We have confirmed that biallelic missense variants may lead to normal NBAS protein levels but markedly reduced p31 levels, indicating protein dysfunction, as previously described.^5^, ^7^ Individual A, with biallelic missense variants, demonstrated normal NBAS protein levels despite evidence of NBAS dysfunction as shown by very low p31 levels. In contrast, individuals B1 and C, who each had a heterozygous missense variant *in trans* with a nonsense variant and deep intronic splicing variant, respectively, had reduced NBAS levels and reduced p31 levels. These results highlight the importance of selecting the correct functional assay depending on the mutational mechanism of the gene and the variants found, as measurement of NBAS protein levels alone would have been insufficient to allow reclassification of the missense VUS in individual A.

Interestingly, though perhaps not unsurprisingly, each of the families described in this case series had at least one *NBAS* missense variant affecting the Sec39 domain (Table 1). Variants in the Sec39 domain are more likely to be seen in individuals with a RALF phenotype, while variants in the β‐propeller domain often present with a mixed phenotype.^6^ None of the patients in our case series of individuals with *NBAS*‐related RALF had a variant in the C‐terminal domain, which is more commonly associated with short stature, optic atrophy, and Pelger‐Huët anomaly.^6^

**TABLE 1:** Clinical and molecular characteristics of individuals with *NBAS* variants

Individual	A	B1	B2	C
Alive	Yes	No	No	Yes
Age at last follow‐up	3 years 3 months	NA	NA	9 years
Age at death	NA	1 year 2 months	2 years 9 months	NA
Previously published	No	No	No	Yes^8^
Facial features	No	NK	NK	No
Abnormality of the liver, HP:0001392				
Acute liver failure	Yes	Yes	Yes	Yes
Elevated hepatic transaminase	Yes	Yes	Yes	Yes
Age at onset of first ALF / ELT	8 months	7 months	NK	13 months
Growth abnormality, HP:0001507				
Intrauterine growth retardation	No	Yes	NK	No
Short stature	No	Yes	NK	No
Abnormality of the nervous system, HP:0000707				
Motor delay	No	No	No	No
Intellectual disability	No	No	No	No
Other		Irritable but well until 7 months		
Skeletal system, HP:0000924				
Reduced bone mineral density	No	NK	NK	No
Delayed closure of the anterior fontanelle	No	Yes	NK	NK
Abnormality of the vertebral column	No	NK	NK	NK
Other		Proximal limb shortening		
Abnormality of the musculature, HP:0003011				
Hypotonia	No	No	NK	No
Skeletal muscle atrophy	No	No	NK	No
Abnormality of the eye, HP:0000478				
Optic atrophy	No	NK	NK	No
Abnormality of the skin, HP:0000951				
Cutis laxa	No	NK	NK	No
Abnormality of the immune system, HP:0002715				
Reduced IgG levels	No	NK	NK	No
Reduced NK cell count	NK	NK	NK	NK
Pelger‐Huët anomaly	Occasional	NK	NK	NK
Biopsies/RCE	A liver biopsy was nonspecific with severe diffuse acute pauci‐inflammatory hepatocellular injury, with zonal hepatocyte necrosis.	RCE in liver were deficient for CII and borderline low for all other enzymes.	RCE in liver was borderline low for CIII and normal for all other enzymes.	Liver biopsy during an acute episode showed microvesicular steatosis. RCE in muscle showed low levels of CII + III activities.
Genomic results				
Allele 1	c.2951T>G; p.(Ile984Ser) (Sec39 domain)	c.2951T>G; p.(Ile984Ser) (Sec39 domain)		c.2617C>T p.(Arg873Trp) (Sec39 domain)
Allele 2	c.406A>G; p.(Arg136Gly) (β‐propeller domain)	c.1213C>T; p.(Arg405*)		c.2423+404G>C

Abbreviations: NK, not known; RCE, respiratory chain enzymology.

We note that individuals B1 and B2, and individual C, underwent extensive investigations for a possible mitochondrial disorder before genomic sequencing identified biallelic *NBAS* variants. Indeed, individual B1 had liver respiratory chain enzyme results suggesting complex II deficiency. We reported previously that complex II is more labile than complexes I, IV and citrate synthase in severe liver disease^24^ so caution is needed in interpreting low complex II levels in this scenario, and we regard the low complex II activity in B1 as secondary to liver failure. This emphasizes that *NBAS*‐associated RALF should be considered as a possible differential diagnosis in individuals with a suspected mitochondrial disorder with prominent liver dysfunction.

The three families presented here represent a spectrum of the diagnostic experience, with the first patient (individual A) receiving ultra‐rapid genomic sequencing results which required functional characterization to confirm pathogenicity of a VUS, the fourth patient (individual C) receiving stepwise genetic results until a molecular diagnosis was achieved, and the second family (individuals B1 and B2) receiving molecular confirmation of a diagnosis over 30 years after death. Ideally, infants and children presenting with RALF and other conditions suspicious for a monogenic disorder should be able to access rapid genomic testing followed by timely adjunct studies (if required) to allow reclassification of VUS in a clinically relevant timeframe. A timely diagnosis of *NBAS*‐associated RALF can encourage aggressive management of acute presentations, potentially obviating the need for liver transplantation. Currently, these adjunct functional studies are not readily available, are usually performed as part of a research study, are time consuming, are not adequately funded, and rely on the good will of the researchers and clinicians involved. Further, VUS are more frequent in ethnically diverse populations that are underrepresented in large population databases,^25^ which further increases inequity. We therefore recommend increasing the availability and funding for timely supportive studies, including RNA, protein, and biochemical studies, to provide functional evidence supporting pathogenicity for VUS detected by genomic sequencing. This will require developing standards and guidelines for integration of functional genomic data into curation pathways and the development of flexible funding models.^26^

There are no published management guidelines for *NBAS*‐related RALF. Based on published case reports and series', *NBAS*‐related acute liver failure is usually self‐limiting and resolves with conservative and supportive management in most,^7^, ^27^, ^28^, ^29^ but not all^30^ individuals. Invasive investigations such as liver biopsy are generally not required. Confirmatory diagnosis of *NBAS*‐related RALF may provide reassurance to treating clinicians and avoid invasive or expensive investigations.

In summary, adjunct functional studies have an important role in assisting with reclassification of VUS identified by genomic sequencing. Funding models for genomic sequencing services should consider incorporating capabilities for adjunct functional studies as part of their service.

## CONFLICT OF INTEREST

All authors declare that they have no conflict of interest.

## ETHICS APPROVAL AND PATIENT CONSENT

All procedures followed were in accordance with the ethical standards of the responsible committee on human experimentation and with the Helsinki Declaration of 1975, as revised in 2000 Parents provided written informed consent for participation in the study. Human research ethics committee approvals were obtained from the Royal Children's Hospital Human Research Ethics Committee (HREC/16/RCHM/150, HREC36291A, HREC 2016.224 [Individual A]) and the Children's Hospital at Westmead (HREC 10/CHW/114 [Individuals B1 and B2] and #10/CHW/113 [Individual C]). Parents provided written informed consent for participation in the study.

## Data Availability

The data that support the findings of this study are available on request from the corresponding author. The data are not publicly available due to privacy or ethical restrictions.
